# Saving sheep – On extinction narratives in Namibian Swakara farming

**DOI:** 10.1017/ext.2024.2

**Published:** 2024-03-07

**Authors:** Eleanor Schaumann

**Affiliations:** Faculty of Humanities and Social Sciences, Cultural and Social Anthropology, University of Bayreuth, Bayreuth, Germany

**Keywords:** Agriculture, Colonization, Death, Drought, Extinction

## Abstract

The Namibian Swakara industry, a type of sheep farming focused on the production of lamb pelts for the fashion industry, currently faces a crisis situation. Formerly one of the most important export products from Namibia, a combination of drought, falling pelt prices and the effects of the COVID-19 pandemic now threaten the survival of Swakara, the Namibian Karakul. The current crisis is articulated in extinction narratives. The potential end of Swakara farming as a way of life and a set of knowledge practices is narratively interwoven with the potential disappearance of Swakara from the Namibian landscape. Extinction narratives in the context of Swakara farming in Namibia blur the lines of human and nonhuman ways of life and their disappearance.

## Impact statement

This article relates key concepts from extinction studies to the context of Swakara farming in Namibia. It demonstrates the relevance of the concepts of ‘double death’ and extinction as an ‘unravelling of a way of life’ to the current crisis situation of a sheep farming sector. In doing so, it extends this theoretical framework to previously understudied fields of extinction in relation to domestic animals and to the overlap of extinction narratives and settler colonial identities in a postcolonial context. Debates around mass extinction tend to foreground the extinction of charismatic wildlife species, while the disappearance of domestic breeds remains understudied. Sheep as a species are not endangered, yet the disappearance of breeds is an issue of concern in regard to agricultural biodiversity. At the same time, it is important not to romanticise these specific ways of farming. This article addresses and complicates these issues.

## Introduction

“It is sad that Swakara is disappearing, because Swakara is a part of us” said Christine, who was married to David,^1^ a white commercial farmer and Swakara stud breeder in southern Namibia. I was visiting the family in late 2020. It had been a year of crisis and unravelling that had hit the Swakara sheep farming sector hard. After 7–8 years of drought and lean sales, the numbers of both Swakara sheep and Swakara farmers were in decline. Her statement illustrates the anxieties over the potential disappearance of Swakara sheep and its implications for the identities and relations of the farming contexts they were situated within.

Many of the remaining Swakara farmers referred to their activities as practices of combatting extinction and preserving a way of life. In this article, I relate these narratives to theoretical frameworks of interdisciplinary extinction studies. In particular, I use the understanding of extinction as an unravelling of a way of life (van Dooren, 2014, p. 7) to make sense of the disentangling and remodelling of multispecies relations in Namibian Swakara farming. I argue that extinction narratives in the storytelling practices of the Swakara industry blur the lines of human and nonhuman ways of life and their disappearance. Endangered animals and endangered knowledges, the presence of a population of sheep in the Namibian landscape and the farmers who care for and kill them, all of these are narratively woven together. The death of individual sheep and the (potential) death of the industry cause rippling effects through the ways of living and farming in southern Namibia. In these processes, meaning-making practices and personal identities are rearranged.

This article is based on ethnographic fieldwork I conducted in Namibia, including two extended periods of participant observation on Swakara farms and at the offices of the Swakara^2^ Board and Breeders’ Society in Namibia in 2020 and 2021, one shorter period in 2022, as well as several interviews with actors in the current and historical Swakara industry. Additionally, I base my argument on primary publications of the industry, such as advertising brochures and the annual reports published by the Karakul Board and the Karakul Breeders’ Society.^3^

The history of Swakara begins with the first Karakul sheep that were brought to Namibia in 1907 at the height of the genocidal war against the Herero and Nama as part of an agricultural experiment of German settler colonialism (Schramm, forthcoming). While Karakul sheep can be used for meat production, the most important product are the pelts from newborn lambs. These pelts are sold at international fur auctions and used for exclusive high-end fashion design. Karakul sheep originate in Bukhara, in current Uzbekistan. The breed’s hardiness and adaptability to arid climates as well as the German fur trade’s desire to reduce dependence on Czarist Russia for Karakul pelts made it an ideal animal in the eyes of colonial officials (Spitzner, 1962; Declercq, 2016).

It was not until the 1930s under South African rule that Karakul farming in Namibia accelerated in scale. In the 1960s and 1980s, it was one of the most important export industries in Namibia, second only to mining (Viljoen, 2008, p. 99 ff.). In fact, Karakul sheep were often referred to as black diamonds (Bravenboer, 2007, p. 84 ff.; Zondagh, 1990), alluding to their value as luxury export product. In 1970, around 95% of all sheep in Namibia were Karakul, which dropped to only 6% in 1998 (Schoeman, 1998, pp. 125–126).

Karakul farming generated significant wealth for a group of white commercial farmers descended from German and Afrikaans speaking settlers through a combination of subsidies from the apartheid government, the exploitation of African farm labour, the appropriation of farming knowledge and land as well as a booming export market for fur (Moore, 2021; cf. Silvester, 1993).

White commercial farmers profited most; they are, however, not the only farmers who have shaped the Karakul industry and who claim Karakul farming as heritage. Black farmers in communal areas, Affirmative Action Loan Scheme farmers, farm workers and their descendants and, to a lesser degree, resettlement farmers are integral to the Karakul industry, present and past. According to a survey commissioned by the Swakara Board in 2013, 47% of Swakara producers were communal farmers, 45% commercial farmers and only 8% resettlement farmers (Kruger et al., 2013, p. 7). In early 2020, most of my interlocutors estimated that communal farmers made up the majority of producers but accounted for only 20% of pelts produced, which were concentrated in the lower price segment.

In 2012, the name Swakara, which had been the label used to market Namibian Karakul pelts since 1966 (Bravenboer, 2007, p. 165), was officially adopted as the name of the Namiban Karakul breed, thus declaring it a breed endemic to Namibia in a move to distance the industry from Karakul farming practices in Central Asia (Karakul Breeders Society, 2012, p. 19). The change was justified through genetic testing, in which samples from Swakara sheep at the research station were compared with Karakul sheep from the flock in Halle (Germany), finding that Swakara sheep were more closely related to Namibian breeds, such as the Namaqua and Blinkhaar Afrikaner, than to the population of Karakul sheep in Germany (Muchadeyi, 2016).

“I don’t think it [the Swakara breed] will go extinct, but the way of life, the sorting, the description is disappearing.” What is at stake to Jasper, a white Swakara breeder is not just the existence of Swakara sheep in Namibia but it is the loss of a way of farming, a way of life and a way of relating to sheep. Van Dooren encourages us to think of extinction in a similar way, not just as the death of the last individual specimen of a species but as the loss of a way of life, the disappearance of “what it means to be” a member of a specific species (van Dooren, 2014, pp. 10–11).

This understanding of species is deeply embedded in the field of multispecies ethnography. Species are not simply “life forms” but “forms of life” (Helmreich, 2009, p. 8). They are not stable taxonomic categories but relational enactments (Yates-Doerr, 2015, p. 310). This understanding becomes particularly relevant concerning multispecies approaches to extinction.

Deborah Bird Rose coined the term “double death” to describe the dissolving of relations, the end of a continuity of cycles and the foreclosure of the possibility of future generations (Rose, 2006, p. 75). In contrast to this, individual death is an intrinsic aspect of life and ensures the continuity of future generations (Rose, 2012; Rose et al., 2017, p. 10). Swakara farming is always about the killing of sheep. The fur pelts exported are produced by killing newborn lambs within the first 4 days of their life. In contrast, the current disentangling of relations might be the harbinger of a form of double death.

Swakara farming is not the only industry affected by cycles of economic crisis. Crises are an intrinsic feature of life within capitalism, the norm more than the exception. This is especially true of commercial farming enterprises in Namibia, where supposedly successful white commercial agriculture was only ever profitable due to immense state support throughout the German colonial era and under the apartheid regime (Schmokel, 1985). Nevertheless, the combination of intersecting crisis, the disappearance of pelt markets, drought, climate change, challenges to the legitimacy of wealth and landownership, the decline in the numbers of sheep, farmers and breeders and the loss of farming knowledges and skills make the Swakara industry an interesting case to look at the convergence of a disentangling of a specific production chain and the disentangling of a specific set of multispecies relations.

## Extinction of domestic animals

Wildlife animals on the scale of species are what comes to mind most readily when discussing extinction. Most conservation efforts focus on this scale. Charismatic megafauna species such as panda bears, whales or elephants become flagship species to conservation, while less charismatic species are sidelined (Lorimer, 2015). Meanwhile, species as the relevant scale of analysis are rarely questioned.

When concerned with domestic animals, the scale of relevance to extinction is located at the level of breeds rather than species. *Ovis aries*, the domestic sheep is not endangered, neither globally nor in Namibia, but many “traditional,” “rare” breeds are disappearing. According to the FAO, 24% of agricultural breeds worldwide are currently at risk of extinction (FAO, 2019, p. 8).^4^ Thirty-four percent of all sheep breeds worldwide are either endangered or already extinct (FAO, 2019, p. 10). These types of databases show the scale of the problem, but what they fail to appreciate is that what is at stake are not just biological species but unique perspectives, ways of living and making worlds in multispecies communities (Chrulew and Vos, 2019, p. 24).

This does not only affect immediate human animal relations and modes of production. In a similar way, as theorised by multispecies theorists of wildlife extinction, ecologies on a scale way beyond the immediate context are affected. In this case, these ecologies are not only wildlife ecologies, though these are also implicated, but knowledge ecologies, scientific networks and production chains (Rahder, 2020; c.f. Moore, 2015, p. 393). In the case of Swakara farming and marketing, we are witnessing an undoing of relations. The absence of flocks of Swakara sheep roaming the landscape has effects on the multispecies communities inhabiting the area. The connections among designers, artists and Swakara producers are dissolving. The courses teaching shearing, lamb description and slaughtering Swakara sheep are frequently cancelled due to a lack of participants.

Numerous critiques of plantation (i.e. Paredes, 2021; Barua, 2023) and factory style farming (i.e. Fitzgerald, 2003; Stanescu, 2013; Blanchette, 2020) have shown that the disappearance of diversity in crops or breeds in favour of optimised yields or growth rates makes agricultural production and multispecies webs of life in general more susceptible to diseases or weather extremes. Reducing biodiversity in favour of optimised yields comes at the price of resilience. Concerns about biodiversity and the preservation of heritage are two discursive patterns that motivate efforts to combat the extinction of domestic animals. These efforts are commonly found among small-scale, organic, or hobby farmers. Efforts to preserve rare domestic breeds as a form heritage are often embedded within frameworks of nationalism or regionalism. In this line of argument, the loss of a specific breed is also a loss of identity (Kroløkke, 2022). Not only do these animals or crops “belong” into these specific landscapes, they also entangle the human inhabitants of these places in communities of practice. Christine, whose quote opened this article, stated that “Swakara is a part of us.” It follows that when Swakara is lost, this constitutes not only a shift in farming practices but a loss of identity.

## Death of a sheep and the death of an industry

The Swakara industry is built on the killing of lambs. Within the first days of life, Swakara lambs lose their distinctive curl patterns. Therefore, lambs destined for pelt production are killed within 4 days of birth. This practice has received vociferous criticism from animal rights groups such as the Humane Society or PETA (i.e. The Humane Society of the United States, 2000; PETA, 2023). Campaigns against the fur trade in general and Karakul farming in particular included boycotts, media campaigns, and the publication of a video by the Humane Society of the United States depicting the brutal removal of an unborn lamb from a pregnant ewe on a farm in Afghanistan. Distancing the Namibian Karakul industry from these practices was the main motivation for renaming of the breed and industry from Karakul to Swakara. Though the change was legitimised through genetic analysis, it was the farming practices and quality control that made Swakara unique in the eyes of its practitioners.

Furthermore, the Board introduced a code of practice and a set of strict guidelines on how to *humanely* slaughter lambs, which went into effect in 2020. These guidelines included prescribed distances of living lambs to those being slaughtered, the use of electric stunning apparatuses and several other measures to reduce pain and stress to the animals being killed. All producers of Namibian Karakul pelts were obliged to comply with this code of practice. In 2020, the Swakara Board commissioned an industry wide audit to review compliance (Swakara Board of Namibia, 2020). There were controversial opinions among farmers on the degree to which the code of practice truly worked in reducing animal suffering. Overall, the Swakara institutions have dedicated considerable resources to this question. As many farmers and Swakara employees were eager to point out, few other agricultural sectors regulated their slaughtering practices to such a degree. Killing “baby lambs” required an entirely different level of moral justification compared to killing a sheep of 5 months age for mutton production.

The farmers I met made sense of killing, especially the killing of newborn Swakara lambs, in diverse ways but to everyone it was in some way meaningful. In fact, several farmers who left the industry named their difficulties in slaughtering “baby lambs” as part of the reason. “You need to have the stomach for it”, said Jasper’s father concerning his other son, who had ceased to farm with Swakara.

Another white commercial farmer argued that killing for pelts was a necessary sacrifice to keep operations on his farm running, which he argued allowed him to preserve the natural balance and biodiversity on his land. As he explained, the lamb died but the mother lived, the breed lived, he lived and his farm workers lived. In this rendering, individual lambs are sacrificed in order to preserve the way of life on the farm, the overall survival of the Swakara breed and even endangered wildlife. This was echoed by a Black communal farmer, who emphasised that in a drought situation, killing lambs was a way of ensuring the future of the overall flock: “You kill the baby in the drought at least the ewe makes it. If you don’t, they both die.” In summary, the assumption was that the lambs died, so that Swakara as a breed, and industry and a way of life could live.

Killing lambs instead of grown animals is what makes the Swakara industry more drought resilient than other industries. Farmers described the cycles of drought and killing in the following way: in drought times, the flock gets older as all lambs are pelted. When it rains, they can keep more sheep and the flock is rejuvenated.

However, the current crisis threatens to transcend to usual cycles of drought and rain. The intersection of multiple crises: drought, the COVID-19 pandemic, the disruption of supply chains, rising fuel prices and increasing cost of living (and farming) make many fear that a tipping point might be near, from which the Swakara industry would not be able to recover in a meaningful way. Some envision alternative futures for Swakara on a smaller, less organised scale, as a sideline for meat sheep farmers or a hobby for rich pensioners. Others hoped for government support, as the Namibian government had supported the industry through direct and indirect subsidies in the past. In the 2010s, the Namibian government named Swakara an industry of national interest with the potential for growth and value adding, especially in relation to wool (Rothkegel, 2015; Ministry of Industrialisation, Trade and SME Development, 2016). In recent years, there has been less overt involvement of the Namibian government with Swakara, much to the frustration of farmers in the communal and commercial areas alike.

In this situation, all Swakara stud breeders are confronted with the problem that their valued stud animals, the result of decades of upgrading, are without economic value. To all of them, the idea of selling their stud animals or giving them away at meat prices is abhorrent. “Before I decide to give them away, I will kill them” is what a white stud breeder told me in 2022. What is at stake to him is not just the existence of Swakara sheep in Namibia. It is the loss of a way of farming, a way of life and a way of relating to sheep. When efforts of preservation and progress are no longer meaningful, relating to the soon to be absent becomes an issue of ‘dying well’ (cf. van Dooren, 2014; Parreñas 2018).

One of the ironies of the current industry crisis was that the shift to meat sheep farming meant that the newborn lambs were no longer being killed, at least not immediately. Meat sheep are only slaughtered at an age of at least 5 months, for Swakara, with its lower growth rates that can easily be 7–8 months. Previously, the lambs died so that Swakara could live, but in the current situation, Swakara was dying because the lambs were not being killed (yet).

In the industry crisis, a shift in the scale of death occurs. It is now about the survival of the breed. However, Swakara is not only a breed but also a marketing brand, a label under which Swakara pelts are marketed globally, while pelts from Central Asia continue to be marketed as Karakul, sometimes within the same auction. Therefore, when considering the potential disappearance of Swakara, it is important to examine its place in global market relations and the (potential) disappearance of markets can lead to the disappearance of a sheep breed.

## Disappearance of a market

Theorists in economics, sociology and anthropology have argued that markets are not *natural* occurrences but are made and maintained through human activity (i.e. Callon, 1998; Tsing, 2009; Graeber, 2011). Market-making practices are “often contested, interrupted, and precarious” (Ouma, 2015, p. 15). The “global connections” that entangle actors in supply chains and markets (Tsing, 2005) are in need of constant maintenance. This is especially true for the Swakara industry which has been undergoing a decline in the market for its core product, the Swakara pelts. Industry institutions have been struggling to maintain the connections, often on a personal level, to fashion designers, potential buyers of skins and other actors in the production chain of the fashion industry, of which they form a part. These efforts go beyond mere advertising or marketing activities.

Swakara pelt prices had not been sufficient to make up for the losses of the drought even before the COVID-19 pandemic hit in 2020, interrupting the transport of pelts from Namibia to Copenhagen. The low prices for Swakara skins are caused by a combination of factors: general developments within the fur industry, animal rights activism, the availability of synthetic fibre and the economy of scale (Nehoya et al., 2022). Several of these factors mutually re-enforced each other, leading to a feedback loop of decline. With falling prices, less farmers produced pelts, leading to a further drop in numbers.

To make it worth producing a line of garments, designers require a certain number of pelts of similar pattern, colour and quality. With the number of pelts falling below a critical level, it is increasingly difficult to fill auction lots with sufficiently similar pelts. Furthermore, the falling number of pelts results in a rise in marketing costs per pelt, as the marketing, sorting and shipping infrastructure involves many fixed costs. “It is like it is on a downward slope and we somehow need to stop it” said a Swakara employee at the pelt centre in Windhoek in early 2020.

The fall of pelt prices occurred both slowly and rapidly. The auctions in 2018 and 2019 produced barely acceptable prices, though the industry was already facing troubling times due to drought and the prices were not enough to make up for rising costs and difficulties of producing. The outbreak of the COVID-19 pandemic led to a total collapse in prices leaving many pelts unsold.

In November 2020, news of the Danish government’s decision to cull the entire Danish mink population in order to curb the spread of a new variant of the SARS-CoV-2 virus hit international headlines. Shortly after, the Danish fur auction house Kopenhagen Fur announced its planned liquidation by the end of 2023 (Al Jazeera, 2020; Buttler and Wienberg, 2020). Until then they would sell off the remaining mink pelts in storage. The news immediately circulated among Swakara farmers via social media and phone calls. Kopenhagen Fur was not only the main seller of Danish mink but also the auction house where the entire production of Swakara pelts was marketed in two auctions per year. “None of us expected Kopenhagen Fur to be the thing that breaks. That something that has been there for so long can just disappear”, “that’s it, now Swakara is finished” were some of the responses of people I interacted with at the time.

In early 2022, some farmers’ pelts had remained unsold at the auctions since early 2020. Waiting for 2 years, only to receive little money at the auction, placed a significant financial burden on farmers. This together with the anticipated liquidation of Kopenhagen Fur meant that farmers no longer trusted in pelt production for income. “No one in their right mind is producing pelts right now,” explained Kobus, a white commercial farmer in 2021.

When considering endangered wildlife species, their worthiness to be preserved is usually evaluated according to their ecological functions or plain charisma. With domestic animals, especially livestock, the use of an animal is directly linked to its chances of surviving. The German word for domestic animal *Nutztier* – use animal, illustrates this. Therefore, in order to preserve a use animal, it needs to be *of use.* Whether Swakara sheep are ecologically useful is highly debatable. In fact, many farms in southern Namibia bear the scars from overgrazing during the industry’s boom years, places where little or nothing grows and dead patches of land.

No one producing pelts presented a problem to the industry at a fundamental level. Pelt production made up the core product that the Swakara breed was optimised for and that tied together the narratives and knowledge practices of the industry. What use is a pelt sheep if no one is buying pelts? In the absence of ecological necessity, those invested in saving the Swakara breed believed that they needed to diversify their farming operations, raise other animals and market other products besides pelts.

## Meat: The sheep of the future

Meat sheep farming was frequently invoked as both a means of survival and the potential end of Swakara. “Back in the 90s, the housewives saved Karakul.” This statement of a white commercial farmer near Grünau referred to the previous crisis of the industry. He claimed that what had allowed the industry to re-emerge after the first collapse of pelt prices and production was the fact that farmer’s wives had insisted on not selling all Karakul sheep but keeping some for personal consumption. That way, when prices started improving in the mid-2000s, several farmers still had small purebred Karakul flocks. Thus, there was still a certain degree of genetic diversity within the Swakara breed’s population which made it possible for farmers to restock and increase the scale of pelt production again. In this framing, it was Karakul meat that had ensured the continued presence of Karakul sheep on farms, which allowed pelt production to re-emerge. Back then, the flocks of Karakul ewes kept for the production of meat for private consumption created the conditions for a revival of Karakul fur production. On the other hand, in 2021, many farmers saw meat sheep farming as a replacement for Swakara. Among great scepticism concerning pelt markets, meat promised a more stable way forward.

The Swakara breed is not well suited for meat production. Focusing on meat production meant a radical shift in breeding direction away from the characteristics that defined the breed. The fastest way to achieve this was by crossbreeding Swakara ewes with meat sheep breeds such as the Dorper or Meatmaster breeds. Among different farmers, there was some ambivalence on whether Swakara as a meat sheep would still be Swakara. At his office in Karasburg, Carl, a white commercial and former Swakara farmer, explained this change in the following way. “The thing is Swakara as mutton doesn’t work. We are starting to genetically change Swakara, focus on mutton, less on pelt qualities. That will take 10 years or so. And it means we lose the thing that makes Swakara.”

The thing that makes Swakara and its potential loss were present themes in how farmers made sense of the current shifts. They were focused around two interrelated things that were changing or disappearing: the knowledge practices of farming with Swakara and the disappearance of the breed as genetic resource, as the presence of a population of genetically and phenotypically defined Swakara sheep in the Namibian landscape. This is illustrated by Carl’s quote. To him, the thing that makes Swakara is not merely about having sheep that are descended by and traceable to Karakul sheep. If they no longer look like Swakara. If lambs are born with ordinary sheep skin, not prized Swakara pelts, then what makes them Swakara is lost.

As of 2022, there were only eight active stud breeders left in the Swakara industry, seven white commercial farmers and the government research station at Gellap Ost. The remaining breeders tended to see themselves as custodians of the genetic diversity of the Swakara breed, as those who need to keep it alive, so that if pelt prices recovered, all the farmers who were currently crossbreeding with meat sheep could return to Swakara pelt production within a few sheep generations. Using Swakara for meat production with pelts as a byproduct was not a viable option in the long term. Shifting to meat production, at least in the large-scale ranching-based farming systems of commercial farms, meant shifting to other sheep breeds.

To Swakara stud breeders, the genetics of the breed were a treasure, the result of generations of labour and dedication. The talk of genetics was pervasive in the industry. Advertisements for stud animals would constantly refer to “the good genetics” of the animals for sale from a particular stud. In a way that greatly resembles Sarah Franklin’s concept of “breedwealth” (Franklin, 2007) the good genetics of a farmers flock were one of the things others would refer to in order to describe their wealth, privilege or future prospects at the fur auctions.

Other farmers, especially those who had mostly left Swakara farming and just kept a small number of sheep for personal nostalgia, were more critical of the straightforward idea of good genetics. According to Carl, the industry had been too focused on producing pelts and neglected all other factors. “We focused too much on pelts and lost the hardiness.” This scepticism of Swakara stud breeding was not a scepticism against there being Swakara in Namibia because Carl followed up on this, saying that nature would quickly fix these mistakes and that the Swakara of the future would be more of a meat sheep but would regain its hardiness and maybe some of its usefulness. It might not be the Swakara of his childhood and he was at peace with that, though he added that he would forever mourn it.

With the declining population of Swakara sheep, inbreeding was on the rise. In 2020, David tried to establish a consortium of the remaining breeders in order to pool the remaining genetic resources and make previously incompatible stud flocks compatible through targeted breeding. This was never realised. Neither was the NaSwa project, which envisioned a joint conservation flock including genetic material of all the remaining Swakara studs.

Ideas to use technologies such as freezing Swakara sperm or genetic material were not seriously considered, as they were deemed too costly. The research station manager, stud breeders and several pelt producers all identified a 4- to 5-year window of opportunity for the survival of Swakara, for pelt prices to improve. Within that time, farmers would still have a flock of purebred Swakara ewes, so that it would be relatively easy to return to pelt production should prices recover. Provided that there were still stud breeders around to provide breeding rams. After this time, the purebred Swakara ewes would be too old to reproduce, and if the meat sheep breeding trend continued, that would be the point where Swakara would have effectively disappeared from the landscape.

The way people narrated this window of opportunity echoed the way they talked about the survival of knowledge. Younger farmers, communal and commercial alike, preferred farming with other animals, cattle, goats, game or meat sheep according to their circumstances. Various actors within the industry talked of the ageing population of Swakara farmers as if they were themselves an endangered species. The old guys who were keeping the industry alive were dying or retiring. As long as these people with the necessary skills were still alive, the industry could be revived, but after a certain time without a new younger generation taking over, it would mean the end. Individual deaths of farmers and Swakara experts coincide with the end of intergenerational continuities, something that is articulated through narratives of endangerment.

## Narratives of an endangered way of life

“This way of thinking is dying out, going extinct. I am the last one,” said Jasper while he explained his breeding strategy to me. In part he was referring to the fact that he was the only remaining Swakara breeder in his wider area. He was also alluding to the fact that his unique approach to breeding and lamb selection would end with him, as there was no one to take over the farm after him.

Farmers and others involved in the industry would seamlessly segue from endangered animals to their endangered trade or lifestyle. Some farmers like Jasper explicitly used the language of extinction when talking about the future of their trade. Others employed a similar language of death and dying when talking about the Swakara industry or even farming in general. “The drought killed us,” “The TV killed farming” or “Brigitte Bardot killed Swakara.” “The whole Swakara thing is systematically dying out” explained Christian, a Black farmer in the communal area near Keetmanshoop. The Swakara thing to him encompassed the presence of Swakara sheep, as well as the knowledge of the trade and the community of Swakara farmers. He continued that “at least my grandchildren will still be able to see what a Swakara ram looks like.” The future he envisioned for his children and grandchildren entailed getting an education and well-paying jobs in town, not farming in the communal area. His grandchildren would not grow up learning the Swakara trade the way he had, but they would still know what this sheep, he considered their heritage, looked like.

Many Swakara farmers would refer to their genealogies of knowledge, fathers, grandfathers^5^ or mentors who taught them all there is to know about Swakara farming. With farmers, as with sheep, their knowledge would supposedly live on after their death through their material and epistemic legacies to their descendants. When descendants sold the sheep and farms and when no one was interested in learning the trade, this continuity was threatened. As with keystone species, people in the Swakara industry would talk of some farmers as keystone farmers. These were people with large flocks of Swakara sheep, stud breeders who preserved the breed’s genetic diversity and breeding knowledge.

David was one of these. As a fifth generation stud breeder, he was a significant figure in the Swakara industry. The pressure of preserving the industry’s future at times weighed heavily on him and he was aware of the cascading effect it would have if he were to leave the industry, as many others looked to him in making their decisions. In our conversations, he frequently mentioned his desire to do his inheritance justice but that he would not wish it upon his children, even if he was sad to see himself as the last generation. “Only old farmers now farm with Swakara, that means knowledge is a problem.”

Many farmers I spoke to talked about the Swakara industry as a living thing, a collective way of life that was being killed by one factor or another. The scales of collective and individual dying were frequently blurred. Several farmers used the expression “it is killing me” or “it is killing my father” to see what the industry had become. These narratives of death resonate with Deborah Bird Rose’s concept of ‘double death’ (Rose, 2006). What these people describe is not just the death of a sheep or the death of a farmer, but the death of a kind, a continuity that affects farmers’ identities and the multispecies ecologies on their farms.

Narratives of conservation and wildlife preservation have been utilised to reinvent white settler identities in southern Africa, with white farmers positioning themselves as custodians of nature, thus legitimising their ownership of land and presence in the postcolonial nation state (Suzuki, 2017). I argue that a similar tendency can be observed regarding the preservation of domestic species, with white commercial farmers positioning themselves as custodians of Swakara, an embodiment of a sustainable way of life.

Several farmers bemoaned the fact that their grandchildren might not even know what a Swakara ewe looked like. However, this way of living, farming and relating to sheep is not innocent. It is not a pristine *indigenous* practice being destroyed by a colonial “will-to-destruction attacking time and connectivity” (Rose, 2006 pp. 75–76). Swakara farming is, itself, the result of violent, genocidal histories of colonialism, land theft and apartheid. These histories reverberate in the present. Though Namibia is officially designated an “upper-middle income country,” it continues to be one of the most unequal countries in the world in terms of wealth and income (World Bank, 2022). This affects not only monetary wealth but also land. After more than 30 years of Independence and land reform, 70% of Namibia’s commercial farmland continue to be owned by white farmers, the descendants of colonial settlers (Namibian Statistics Agency, 2018, p. 33).

Many descendants of white commercial Karakul farmers were proud to inform me that the entire south of Namibia was “developed with Karakul money.” Several commercial farmers would tell me the origin stories of their families, whose “hard work” made desert land farmable through Karakul (c.f. Swanepoel, 2020, p. 104 ff.). In this narrative, the supposed hardiness of Karakul sheep is conflated with that of white settlers.^6^ These meritocratic development narratives de-emphasise the fact that most of these farms were only able to survive due to government support (Schmokel, 1985; Weigend, 1985).

These narratives were infused with racism against Black Namibians and were used to legitimise the economic, social and land inequalities. Farmers would position themselves as custodians of the land they were farming on, justifying their extractive farming practices with their care for their land’s biodiversity. One farmer related this to the practice of killing newborn lambs. “I have to do it to live, that is the price to keep everything here going.” He then proceeded to list how many people lived off his farm, by which he meant the farm workers he employed at minimum wages and their families. From this, he effortlessly segued into a list of animal and plant species to be found on his land.

“I want to keep the practices alive. Even if we do all crossbreeding, I want to keep doing it this way, gather pregnant ewes together, examine each lamb.” To this farmer, the knowledge of how to describe a lamb, the breeding science and appreciation of the beauty of a Swakara lamb were as important as the genetic resources of the breed. He was not alone in this opinion. Whenever I asked about the future of Swakara, even among stud breeders, the responses always focused primarily on knowledge and pelt prices, only then on genetics and living animals. From this I conclude that the Swakara way of living and farming has value in itself according to the remaining farmers in the industry. It is at least as important as the preservation of the breed as a genetic resource. In face of the potential absence of Swakara sheep, preserving the Swakara way of life becomes a meaning-making practice in its own right.

## Conclusion absence and its anticipation

According to the formal categorisation of the FAO, Swakara is not an endangered breed (FAO, n.d.).^7^ Meanwhile, its potential disappearance was self-evident to my interlocutors in southern Namibia. Many used this disappearance, the anticipation of extinction as a way of deriving meaning from their situation.

Fighting extinction, dedicating one’s life to the preservation of a sheep breed, even if it is a battle that would most likely be futile, was a way for white commercial farmers, Swakara stud breeders in particular, to reframe their identities. As custodians of Swakara knowledge and the Swakara breed, this new settler identity no longer developed and extracted wealth from the land but instead cared for the species inhabiting it, Swakara sheep and wildlife alike. In this, extinction narratives blur the lines of human and nonhuman ways of life and their disappearance.

Extinction occurs at multiple overlapping scales and temporalities. There will probably be Swakara sheep in Namibia for a while yet. At a scale of species, there is nothing at all to be worried about. *O. aries*, the domestic sheep, continues to flourish in southern Namibia. Yet, many farmers believe that in 5 years there might be no more Swakara sheep in Namibia. By this, they do not mean that there will not be any lambs descended from Swakara sheep but that they will not have the curl types and patterns that are essential to the breed and the way of life that they embody.

The lives of different species are not only entangled in immediate ecological cohabitation but also through commodity chains that can span across the planet. The closure of the auction house Kopenhagen fur in Denmark affected the survival of a niche industry in Namibia and thus the survival of the Swakara breed. To the actors within this industry, the survival of the Swakara breed and the survival of their way of life were inseparable. Extinction, in this case, was simultaneously the disappearance of a genetically distinct breed and a specific set of knowledge practices, This not only affected economic income but endangered a white settler colonial farming lifestyle that had been, to a large extent, built around Karakul farming. The ways that these farmers frame their situation as a matter of extinction of a way of life echoes van Dooren’s concept of extinction as the unravelling of a way of life. It may not be innocent, or even likable to many outsiders, but to those within the Swakara industry, the disappearance of ways of knowing, living with and killing Swakara sheep is something to be mourned.

While the slaughtering of newborn lambs became problematic to many of those I talked to, when it occurred on an industrial scale, the fact that lambs were no longer being killed was seen as the death of the Swakara industry, the end of a way of farming, a set of knowledge practices and a way of life. The lambs live, at least for a while, but Swakara, as it was, is dying.

## Data Availability

The data used in this article are not publicly available as it infringes upon the privacy of research participants.

## Footnotes

1 All names of research participants have been changed for the purpose of anonymisation.

2 The name of the Namibian Karakul breed was officially changed to Swakara in 2012. Swakara, an acronym for South West African Karakul, which had previously been used as a fashion marketing brand, was now the name of the breed. The industry institutions changed their names accordingly: the Karakul Board became the Swakara Board, the Karakul Breeders’ Society became the Swakara Breeders’ Society. In this paper I use the term Karakul when referring to the industry in a long-term historical sense. I use Swakara when referring to official activities of the industry institutions after 2012, or when citing informants who used the new names. Many would use both names interchangeably.

3 This work was carried out within the research project “Karakul Circulations” within the Africa Multiple Cluster of Excellence at Bayreuth University. Run by Katharina Schramm and Memory Biwa, our project team combines approaches from history, art and anthropology to trace the circulations of material wealth, power, bodies and knowledges through Karakul sheep as analytic nodal points.

4 This number might well be significantly higher, as there is no current data on 59% of the breeds monitored (FAO, 2019, p. 9).

5 In southern Namibia, as in many other places, farming and farm ownership are highly gendered practices. There were several female Swakara farmers; and women’s labour and knowledges are crucial, though often rendered invisible in the hegemonic narratives of the Swakara industry. Therefore, farming knowledges and farm inheritance were mostly imagined as a transfer from father to son. In light of intersecting crises affecting farming, and many farmers’ difficulties in finding successors, this appears to be in the process of slowly changing.

6 Sarah Franklin observes a similar effect in the imaginary of sheep as hardy, adaptable frontier animals settler colonialism in Australia (Franklin, 2007, pp. 118–120).

7 As of April 2023, the FAO data on the population of Swakara sheep only extended until 2016 (FAO n.d.). Considering the stark decline since 2020, the risk assessment might be different if numbers were more up to date.

## References

[r1] Al Jazeera (2020) World’s biggest fur auction house to close after mink COVID cull. Retrieved January 29, 2022, from https://www.aljazeera.com/economy/2020/11/13/worlds-biggest-fur-auction-house-to-close-after-mink-covid-cull.

[r2] Barua M (2023) Plantationocene: A vegetal geography. Annals of the American Association of Geographers 113(1), 13–29. 10.1080/24694452.2022.2094326.

[r3] Blanchette A (2020) Porkopolis: American Animality, Standardized Life, and the Factory Farm. Durham, NC: Duke University Press. 10.1215/9781478012047.

[r4] Bravenboer B (2007) Karakul: Gift from the Arid Land: Namibia 1907–2007. Windhoek, Namibia: Karakul Board of Namibia : Karakul Breeders’ Society of Namibia.

[r5] Buttler M and Wienberg C (2020, November 13) *World’s Biggest Fur Auction House Plans to Liquidate Assets. Bloomberg.* Retrieved from https://www.bloomberg.com/news/articles/2020-11-12/kopenhagen-fur-to-close-after-denmark-orders-mass-mink-cull.

[r6] Callon M (Ed.) (1998) The Laws of the Markets. Oxford ; Malden, MA: Blackwell Publishers/Sociological Review.

[r7] Chrulew M and Vos RD (2019) Extinction: Stories of unravelling and reworlding. Cultural Studies Review 25(1), 23–28. 10.5130/csr.v25i1.6688.

[r8] Declercq R (2016) Building imperial Frontiers: Business, science and karakul sheep farming in (German) South-West Africa (1903–1930). Journal of Modern European History 14(1), 54–77.

[r9] FAO (2019) Status and trends of Animal Genetic Resources. Commission on Genetic Resources for Food and Agriculture. Retrieved from http://www.fao.org/3/my867en/my867en.pdf.

[r10] FAO (n.d.) Swakara (Karakul)/Namibia (Sheep): Breed data sheet. Retrieved April 19, 2023, from https://www.fao.org/dad-is/browse-by-country-and-species/en/.

[r11] Fitzgerald D (2003) Every Farm a Factory : The Industrial Ideal in American Agriculture. New Haven : Yale University Press.

[r12] Franklin S (2007) Dolly Mixtures: The Remaking of Genealogy. Durham: Duke.

[r13] Graeber D (2011) Debt: The First 5,000 Years. Brooklyn, NY: Melville House.

[r14] Helmreich S (2009) Alien Ocean: Anthropological Voyages in Microbial Seas. Berkeley: University of California Press.

[r15] Karakul Breeders Society (2012) Swakara Annual Report 2012–2013. Swakara Annual Report, 53.

[r16] Kroløkke C (2022) Heritage with cows. Conserving Nordic human and nonhuman animals. International Journal of Heritage Studies 28(8), 955–969. 10.1080/13527258.2022.2099448.

[r17] Kruger B, Hugo P and van Zyl J (2013) The importance of the Namibian Swakara industry and opportunities for growth. Windhoek: agra.

[r18] Lorimer J (2015) Wildlife in the Anthropocene: Conservation after Nature. Minneapolis: University of Minnesota Press.

[r19] Ministry of Industrialisation, Trade and SME Development (2016) Growth strategy for Namibia’s swakara wool industry and associated value chains. Retrieved from https://csnk.cz/wp-content/uploads/2019/02/Swakara-Wool_Strategies_Web.pdf.

[r20] Moore BC (2021) Smuggled sheep, smuggled shepherds: Farm labour transformations in Namibia and the question of Southern Angola, 1933–1975. Journal of Southern African Studies 47(1), 93–125.

[r21] Moore JW (2015) Capitalism in the Web of Life: Ecology and the Accumulation of Capital. London, New York: Verso Books, 336 pages.

[r22] Muchadeyi FC (2016) *Investigating Population Genetic Structure and Genomic Differences between Swakara Sub-Populations: Towards Identification of Causal Mutations and Development of Markers for Sub-Vitality Traits in White Pelt Production.* Agricultural Research Council-Biotechnology Platform.

[r23] Namibian Statistics Agency (2018) Namibia Land Statistics Booklet. Retrieved from www.nsa.org.na.

[r24] Nehoya L-AT, Kalundu K and von Bach HJS (2022) Modelling supply response and volatility of Swakara pelts in Namibia: Karakul, production, trend, autoregressive distributed lag, and sustainability. Welwitschia International Journal of Agricultural Sciences, 3(1), 50–63.

[r25] Ouma S (2015) Assembling Export Markets: The Making and Unmaking of Global Food Connections in West Africa. Chichester, West Sussex, UK; Malden, MA: John Wiley & Sons Inc.

[r51] Parreñas JS (2018) Decolonizing extinction: the work of care in orangutan rehabilitation. Durham: Duke University Press.

[r26] Paredes A (2021) Experimental science for the ‘Bananapocalypse’: Counter politics in the Plantationocene. Ethnos 88(4), 1–27. 10.1080/00141844.2021.1919172.

[r27] PETA (2023, September 7) Lammfell: Werden Schafbabys für das Fell getötet? Retrieved September 24, 2023, from https://www.peta.de/themen/lammfell/.

[r28] Rahder M (2020) An Ecology of Knowledges: Fear, Love, and Technoscience in Guatemalan Forest Conservation. Durham: Duke University Press.

[r29] Rose DB (2006) What if the angel of history were a dog? Cultural Studies Review 12(1), 67–78. 10.5130/csr.v12i1.3414.

[r30] Rose DB (2012) Multispecies knots of ethical time. Environmental Philosophy 9(1), 127–140.

[r31] Rose DB, Van Dooren T and Chrulew M (2017) Introduction. In Extinction Studies: Stories of Time, Death, and Generations. New York: Columbia University Press, pp. 1–18.

[r32] Rothkegel B (2015) The Swakara Industry: Past and present efforts of value addition of pelts and wool.

[r33] Schmokel WW (1985) The myth of the white farmer: Commercial agriculture in Namibia, 1900–1983. International Journal of African Historical Studies 18(1), 93. 10.2307/217975.

[r34] Schoeman SJ (1998) Genetic and environmental factors influencing the quality of pelt traits in karakul sheep. South African Journal of Animal Science 28(3). 10.4314/sajas.v28i3.44243.

[r35] Schramm K (forthcoming) Race, Species, Knowledge: Approaching Coloniality through Karakul Sheep.

[r36] Silvester J (1993) Black Pastoralists, White Farmers: The Dynamics of Land Dispossession and Labour Recruitment in Southern Namibia 1915–1955. London: University of London.

[r37] Spitzner KW (1962) *Die Karakulzucht in Südwestafrika und das Haus Thorer.* Kapstadt.

[r38] Stanescu J (2013) Beyond biopolitics: Animal studies, factory farms, and the advent of Deading life. PhaenEx 8(2), 135. 10.22329/p.v8i2.4090.

[r39] Suzuki Y (2017) The Nature of Whiteness: Race, Animals, and Nation in Zimbabwe. Sivaramakrishnan K (ed). Seattle, London: University of Washington Press.

[r40] Swakara Board of Namibia (2020) *Swakara Code of Practice. Annexure of Factual Findings.* Windhoek.

[r41] Swanepoel J (2020) In the Land of the Jackals: Postcolonial Aridity in Southern Namibia. Bloemfontein: University of the Free State.

[r42] The Humane Society of the United States (2000) Karakul Sheep and Lamb Slaughter for the Fur Trade, Washington. Retrieved from https://www.humanesociety.org/sites/default/files/docs/investigation-report-karakul-sheep-lamb-fur-trade.pdf.

[r43] Tsing AL (2005) Friction: An Ethnography of Global Connection. Princeton, NJ: Princeton University Press.

[r44] Tsing AL (2009) Supply chains and the human condition. Rethinking Marxism 21, 148–176. 10.1080/08935690902743088.

[r45] van Dooren T (2014) Flight Ways: Life and Loss at the Edge of Extinction. New York: Columbia University Press.

[r46] Viljoen J (2008) *Die geskiedenis van karakoelboerdery in Suidwes-Afrika. 1907–1950.* Windhoek.

[r47] Weigend GG (1985) Economic activity patterns in white Namibia. Geographical Review 75(4), 462. 10.2307/214413.

[r48] World Bank (2022) Country Overview: Namibia. (Text/HTML). Retrieved November 15, 2022, from https://www.worldbank.org/en/country/namibia/overview.

[r49] Yates-Doerr E (2015) Does meat come from animals? A multispecies approach to classification and belonging in highland Guatemala. American Ethnologist 42(2), 309–323. 10.1111/amet.12132.

[r50] Zondagh C (1990) *Karakoel: Diamant of Matrys?* Eirup Namibia: s.n.

