# Supplementary material for: Saving sheep – On extinction narratives in Namibian Swakara farming
Source: Camb Prism Extinct. 2024 Mar 7;2:e1. doi: 10.1017/ext.2024.2 (PMC11895743; doi:10.1017/ext.2024.2)
Supplement: Peer review history

# Editor report: Recommendation: Saving sheep – On extinction narratives in Namibian Swakara farming — R0/PR2

**DOI:** 10.1017/ext.2024.2.pr2

**Affiliations:** School of English, University of Leeds, United Kingdom of Great Britain and Northern Ireland

The essay has received two positive reviews, but several minor revisions and clarifications have been requested and these should be completed before the essay is approved for publication. My strong advice as handling editor is that the recommendations of the two reviewers should be followed closely: they are both experts in the field and their reviews are extremely helpful in pointing out the areas where the essay can be improved.

# Editor report: Decision: Saving sheep – On extinction narratives in Namibian Swakara farming — R0/PR3

**DOI:** 10.1017/ext.2024.2.pr3

**Affiliations:** School of Natural Sciences, University of Tasmania, Australia

No accompanying comment.

# Editor report: Recommendation: Saving sheep – On extinction narratives in Namibian Swakara farming — R1/PR5

**DOI:** 10.1017/ext.2024.2.pr5

**Affiliations:** School of English, University of Leeds, United Kingdom of Great Britain and Northern Ireland

No accompanying comment.

# Editor report: Decision: Saving sheep – On extinction narratives in Namibian Swakara farming — R1/PR6

**DOI:** 10.1017/ext.2024.2.pr6

**Affiliations:** School of Natural Sciences, University of Tasmania, Australia

No accompanying comment.
